# Chronic Disease Patients’ Engagement in Interprofessional Telehealth Collaboration in Primary Care: A Scoping Review

**DOI:** 10.1177/21501319251333858

**Published:** 2025-06-06

**Authors:** Monica McGraw, Anaelle Morin, Vanessa Tremblay Vaillancourt, Marie-Eve Poitras, Yves Couturier, Isabelle Gaboury, Marie-Dominique Poirier

**Affiliations:** 1 Université de Sherbrooke Saguenay, QC, Canada; 2 CRMUS Research Chair on Optimal Professional Practices in Primary Care, Saguenay, Canada; 3 Centre intégré universitaire de santé et de services sociaux du Saguenay—Lac-St-Jean, Saguenay, Canada; 4 Université de Sherbrooke, QC, Canada

**Keywords:** primary care, interprofessional collaboration, disease management, tele-medicine, patient-centeredness

## Abstract

With the rise of people being affected with chronic illness, now the leading cause of mortality worldwide, primary care is overwhelmed with the demand for healthcare services. Primary healthcare is the first resource for patients living with chronic illness, but in 2019, COVID-19 brought healthcare professionals to increase services through virtual care for patients living with chronic illness. In the workplace, such professionals often need to be sufficiently resourced to collaborate, to address collaborative care barriers in telehealth and to keep patients engaged in their health. We performed a scoping review to identify how patients living with chronic diseases actively engage and describe their involvement in the process of interprofessional collaboration within the context of telehealth in primary care settings. We followed Arksey and O’Malley’s and the Joanna Briggs Institute’s methodological guidelines to conduct this scoping review. The analysis of the retained twelve studies showed little distinction between the experience of interprofessional collaboration from the patient’s perspective in a telehealth context compared to a face-to-face context. However, we were able to identify gaps (eg, limited insight onto engagement dynamic, lack of patient-centric research, and insufficient research on patient engagement) relating to the experiences of patients, patient engagement, and professionals who have used telehealth. In an era of digital innovations, this lack of literature regarding the patient experience may jeopardize the quality of the interprofessional collaboration services offered to patients and patient engagement. This gap in patient engagement integrated into interprofessional collaboration in a telehealth context needs to be addressed.

## Introduction

Telehealth has been defined as “the delivery of healthcare and related clinical/non-clinical services such as patient-to-provider interaction, provider-to-provider interaction, healthcare education, self-care, and health information services remotely using telecommunication technologies such as computers, mobile devices, and wearable devices.”^1^ It is one of many innovative practices that were implemented quickly in the healthcare system and without infrastructure.^2^ This care modality has been around for many years in the healthcare system and primary care; yet its use still faces certain challenges (eg, technology access and literacy; data privacy; and limited physical examination capabilities).^3-5^

We could emphasize that telehealth fundamentally changes the dynamics of communication and the environment in which collaboration and patient engagement occur. Unlike in-person care, telehealth limits the non-verbal cues and spontaneity of exchanges among healthcare professionals and patients, which can affect the quality and immediacy of interprofessional collaboration.^3-5^ Additionally, telehealth relies heavily on technology, which may introduce accessibility issues or technical barriers that can hinder the level of patient engagement and team interaction.^6,7^ These factors make it essential to investigate how interprofessional collaboration and patient engagement adapt or are limited in telehealth settings. This underscores the need for targeted strategies to enhance both elements in virtual care environments.

We need a successful healthcare professional team to deliver great telehealth services. To ensure the success of the healthcare professional team, the professional needs to integrate interprofessional collaboration and patients in their everyday practice.^6^ This type of collaboration must also be present in the telehealth context.^7^ It is often defined as “a practice that occurs when several health and social services professionals cooperate with colleagues, patients, and/or their loved ones to provide the highest possible quality of care in different intervention settings.”^8^

Interprofessional collaboration is beneficial for a patient living with chronic diseases.^9,10^ The benefits of interprofessional collaboration also include increased job satisfaction and motivation among professionals,^11^ practice quality,^12,13^ a stronger patient engagement in care,^10^ and as a result, a more positive experience of care and, therefore, of their health.^14^

Patient engagement is an important, new, and essential component to be integrated into the interprofessional collaboration for patients living with chronic diseases.^9,10^ This concept goes beyond patient satisfaction with care and implies the participation and involvement of the patient in their care according to what they want and can do, in partnership with their healthcare provider and integrating personalization, access, engagement, and therapeutic alliance.^10^

## Background

Recently, patient care delivery has undergone significant changes and challenges in accessibility. Primary care is the main entrance to healthcare for people with chronic diseases.^15-17^ The increasing prevalence of aging populations and individuals living with chronic diseases has become evident to both governmental bodies and primary care practitioners.^18^ Accessibility to primary care is getting more complicated due to the complexification of patient conditions and the rise in demand for healthcare.^17,18^ When the global pandemic arrived in 2020, the accessibility to healthcare was even more of a challenge than before in Canada. This brought a rise in telehealth use and has led to varying levels of social distancing restrictions worldwide.^16^ Mandatory public health measures included a restriction on public gatherings, the operation of business, and, most importantly, the accessibility of healthcare.^18,19^ In response to challenges in accessing primary healthcare, the Minister of Health pushed the implementation of telehealth.^12,19^ Several global health authorities wanted to keep access to primary care for patients with chronic diseases during this period of lockdown, followed by a period of social restriction, all while implementing telehealth to ensure accessibility.^16,19^ However, to maintain this form of accessibility for healthcare and ensure its sustainability, the patient’s experience needed to be assessed. There is little literature on the experience of patients living with chronic diseases in telehealth, including how the patients are engaged in telehealth.^4,12,16^

The existing literature does not adequately cover the experience of patient engagement with interprofessional collaboration, particularly in telehealth contexts where patients are actively engaged.^17,20,21^ Although literature abounds with the benefits of telehealth being implemented and improving access to the healthcare system, there is mainly limited information regarding the experience of patient engagement in an interprofessional collaboration process.^4,12,16,17,20-22^ To provide high-quality care grounded in patient experience, we must understand what exists in the literature regarding patient engagement in the context of collaborative telehealth.

To address this knowledge gap, we conducted a rapid scoping review utilizing the meta-aggregation methods^23^ with the following objectives: explore the scope of the experience of patient engagement in the interprofessional collaboration process while utilizing telehealth during a primary care appointment.

## Method

This rapid scoping review was conducted under the Joanna Briggs Institute’s methodology for scoping reviews,^24^ which is based on previous work by Peters et al^24^ and Arksey and O’Malley.^25^ This method is the most appropriate to examine the literature comprehensively. We reported our results using the Preferred Reporting Items for Systematic reviews and Meta-Analyses extension for Scoping Reviews (PRISMA-ScR).^23^ A rapid scoping review is especially useful for quickly mapping the landscape of research in a specific field, offering a broader overview without the extensive detail characteristic of a systematic review.^23^ This approach is less time-constrained and allows for the swift transfer of knowledge to support both practice and policy, especially in rapidly evolving contexts such as those involving telehealth, interprofessional collaboration, and patient engagement.

## The Research Question

This rapid scoping review was guided by the question: How do patients living with chronic diseases describe their engagement in the interprofessional collaboration process in a telehealth context in primary care settings?

## Data Sources and Search Strategy

To identify relevant studies, the search was assisted by an experienced librarian at the University of Sherbrooke (Canada). An initial exploratory search was conducted using MEDLINE to validate search terms contained in relevant articles to develop a full search strategy. The search terms and strategy were validated through input from the research team and the experienced research librarian. Disagreements between reviewers were resolved through a consensus approach. When there was a discrepancy, the reviewers engaged in discussions where they critically evaluated the citations and collaboratively agreed on the final inclusion or exclusion of studies. When a disagreement arose, the 2 reviewers revisited the inclusion or exclusion criteria one by one until reaching a consensus based on the content of the article. The roles of the reviewers were clearly delineated, with 1 reviewer initially taking the lead in the screening process, while the other acted as a backup reviewer, ensuring consistency and reducing bias through a second, more cautious evaluation. Published literature between January 2002 and September 2023 was conducted using the following electronic databases: PubMed, Cochrane, and EBSCO (MEDLINE and Cumulative Index to Nursing and Allied Health Literature [CINAHL]). We used the filter to only keep 20 years of literature starting in 2002, given that telehealth had been used before the arrival of COVID-19 in primary care teams and was starting to have an impact on the healthcare system.^5,17^ Secondly, the search strategy was pilot-tested and refined to compile a list of keywords from titles, abstracts, and MeSH terms used in most relevant publications to the review. All identified key terms in grey literature were consulted using Google Scholar, Google, Grey Literature Report, and Conference Proceedings to identify studies, reports and abstracts relevant to this review. The search strategy included keywords (Supplemental File) associated with the following concepts: (1) Chronic diseases; (2) Interprofessional collaboration; (3) Patient engagement; (4) Primary care, and (5) Telehealth. Additionally, the reference list of included studies were hand-searched to identify more relevant literature. Studies were included if they were published

(1) in French or English.
(2) between January 2002 and September 2023.
(3) articles who contributed to answering the research question.
(4) articles that used a quantitative, qualitative, mixed methods design as well as literature reviews.
(5) articles involving patients without chronic illnesses, as these studies offered relevant insights into other key concepts critical to our analysis.

## Charting the Data

Following the search, all identified records were collated and uploaded into Zotero, and duplicates were removed.^26^ After the removal of duplicates, all the citations were uploaded to Covidence.^27^ Then 2 independent reviewers (from among MM and AM) independently screened the title and abstract of each citation and selected studies for assessment against the inclusion criteria for the review. Discrepancies were resolved through a review process where the 2 reviewers compared their extractions side by side, discussed any differences, and reached consensus on the final data entered the analysis.

For data analysis, we used thematic analysis to systematically identify and interpret patterns across the selected articles.^28^ We extracted data primarily from the results, discussion, and conclusion sections of each article to capture both the empirical findings and the authors’ interpretations related to patient engagement and interprofessional collaboration in a telehealth context.

The thematic analysis involved an iterative coding process. Initially, we reviewed the articles and assigned codes to specific text segments, focusing on themes related to patient perspectives, collaboration processes, and telehealth implementation. As patterns emerged, we refined these codes into broader themes. Multiple researchers (MM, AM, MEP, IG, and YC) were involved in the theme development, promoting consistency and reducing potential bias through collaborative discussion and review. Any disagreements in theme interpretation were resolved through consensus meetings, where we critically evaluated differing perspectives until agreement was reached.

In studies with quantitative data, we integrated these findings by aligning them with the qualitative themes, comparing trends, and outcomes where applicable to add depth and context. This mixed-methods integration allowed us to capture a comprehensive view, ensuring that both quantitative indicators and qualitative insights contributed to the final thematic framework.

## Results

**Figure 1.**
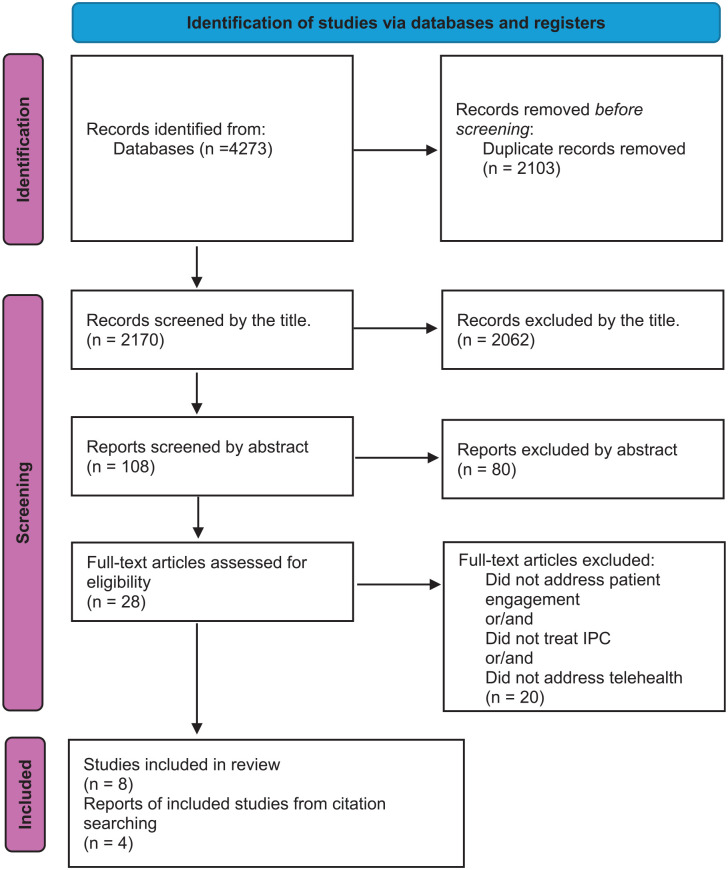
Number of documents in the search strategy.

The search strategy identified a total of 4273 articles through database searches. After extracting the duplicate (2103), a total of 2170 articles were screened based on subsequent exclusion by title based on predefined criteria reducing the number to 108 articles. Further scrutiny involving the exclusion by title and summary articles led to the elimination of an additional 80 articles. A meticulous review of the full text resulted in the exclusion of 20 more articles, leaving 8 articles for the final analysis. To enhance the comprehensiveness of the review, the study incorporated gray literature and snowballing methods,^29,30^ adding 4 articles from these sources. Consequently, the total number of included articles increased to 12 (Table 1). To illustrate the search and selection process during the scoping review, we used the PRISMA-ScR diagram shown in Figure 1. The inclusion process ensured a robust and focused selection of articles, providing a comprehensive overview of interprofessional collaboration interventions and their impact on the experience of patient engagement. Of the included articles, 75% were published after 2021, as shown in Table 1. Studies took place in a variety of locations (Canada, the USA, Netherlands, the UK, Sweden). All articles were published in English.

**Table 1.** Description of Included Studies.

Authors and Country of the study	Design and Population	State of knowledge
Agarwal et al. (2022) Canada	Cross-sectional study 7532 patients University Medical Clinic	85% received timely care 93% included in decision-making 92% are highly satisfied with the care they receive 78% want to continue telephone consultations Future: integrating the patient experience
Arnaert et al. (2022) Canada	Qualitative descriptive study 22 post-bariatric surgery patients	Satisfaction with access to healthcare professionals Redundancy of information during telehealth consultations Need for a structure on the use of telehealth Future: patient commitment and motivation required
Barenfeld et al. (2022) Sweden	Mixed study 86 patients	Satisfaction good Communication gaps (less contact, non-verbal, etc.) The use of telehealth facilitates the patient-professional partnership in the implementation of care plans
Baughman et al. (2022) Pennsylvania and Maryland	Retrospective cohort study 526,874 patients (409,732 in office: 117,142 telemedicine)	Telemedicine (video) Patients exposed to telemedicine had comparable or better performance on most quality measures Care quality
Breton et al. (2021a) Canada	Transversal study 603 primary healthcare professionals	Travel reduction Improving efficiency Reduced risk of infection transmission Difficulty developing relationships with patients
Breton et al. (2021b) Canada and Massachusetts	Comparative qualitative study 20 Doctors Quebec 22 Doctors Massachusetts	Accessibility Less travel Suitable for patients’ work/appointment schedules Technological barrier Professional impact Flexibility Difficult teamwork Technological barrier Efficiency Quicker Fewer cancellations Difficulty of assessment Relational dimensions of care Easy communication Difficult therapeutic relationship Reduced patient involvement
Brodar et al. (2021) United States	Quality improvement approach 2 Psychologist, 3 psychologist student, 2 diabetes educator, and 1endocrinologist	Lack of a model for telehealth consultation and liaison Reduced interdisciplinary collaboration Reduced patient commitment Reduced public activity
Cartwright et al. (2013) United Kingdom	Pragmatic cluster randomized controlled trial 1,573 patients with COPD, diabetes, or heart problems between May 2008 and December 2009	Telehealth without infrastructure increases the complexity of patients developing a therapeutic relationship with a professional Communication is disjointed Reduced quality of care if telehealth is not used properly
Overend et al. (2018) Canada	Pilot study of an intervention 53 patients—Interior Health Authority (IHA)	82% satisfaction 62% want to continue telehealth Telehealth is considered an important consultation method for vulnerable patients
Poitras et al. (2021) Canada	Qualitative descriptive study 40 Patients (active FMG) living with a chronic illness and/or mental health condition. Patients with a follow-up by a doctor, nurse, social worker, nurse practitioner	Satisfaction from patient using telehealth Quicker, easier access to the doctor Time saved Follow-up is more difficult with social worker and health professionals, or the relationship was not already made Fewer follow-up requests, as patients try to cooperate autonomously before asking for a follow-up Most of the information retrieved is part of the cooperative strategies of self-care
Poitras et al. (2024) USA and Canada	Scoping review Health and Social care professionals 31 articles 17 websites and government documents	Patient perception Patient satisfaction Accessibility Self-care Telehealth—mandatory Positive adaptation Strategies for Telemedicine Maintaining optimal health Hobbies Family and social relations Work Negative coping strategy Lack of time Stress Fast food consumption Antidepressants Anxiety
Van Gurp et al. (2016) Netherlands	Qualitative study 18 patients	Enables continuity of care thanks to faster connectivity Beneficial for patients with physical complexity Professional disengagement Working in silos Potential to facilitate interprofessional The lack of structure slows down the process of using telehealth

Current literature lacks a direct exploration of how telehealth uniquely influences interprofessional dynamics and patient engagement, both essential for effective care. The review reported little difference between telehealth and in-person contexts. Not all articles specifically offered insights from patients living with chronic illnesses; however, they were selected for their relevance to other key concepts. These articles contributed significantly to theme identification and provided valuable information for the study. We were able to identify 3 themes relating to patient engagement, the experience of patients and professionals who have used telehealth. The articles included information according to these themes; (1) Advantages of interprofessional collaboration using telehealth in primary care; (2) Challenges of telehealth in primary care; (3) The importance of suitable use of telehealth to maintain patient engagement and interprofessional collaboration in primary care.

## Theme 1: Advantages of Interprofessional Collaboration Using Telehealth in Primary Care

### Improve Quality of Care

Interprofessional collaboration is a process often associated with an improved quality of care (eg, accessibility; patient satisfaction, patient engagement, etc.) that is delivered by healthcare professionals.^4,7^ Patients will often talk about their experiences to provide information regarding the quality of care from their healthcare professional.^5,31^ As Poitras et al,^4^ notes, “*effective interprofessional collaboration enhances patient satisfaction and fosters a deeper sense of involvement in care decisions, contributing to overall better health outcomes*.”

### Maintaining Professional Relationships

Interprofessional collaboration allows an interprofessional working relationship to be maintained between team members and the patient either in an in-person or virtual community, which in turn enables optimal health outcomes to be achieved. According to several authors, interprofessional collaboration through telehealth contributes to increasing job satisfaction among professionals, as well as improving quality and clinical practice.^7,32^

### Patient Engagement and Chronic Disease Management

Poitras et al,^4^ highlight the importance of interprofessional collaboration in improving engagement among patients living with chronic diseases. Patient engagement is conceived as an integral part of interprofessional collaboration (eg, developing care plans with the team, participating in treatment choices, etc.), enabling patients living with chronic diseases to participate actively in decision-making about their health.^22,31^ For these patients, one of the advantages of telehealth is the facilitated access to several professionals which enhances the services received.^4^ Studies show that patients living with chronic diseases are increasingly interested in and/or desire accessibility to telehealth services as an alternative modality to face-to-face appointments.^3^ This alternative modality is very important information because it will prevent difficult travel for them.

### Enhancing Collaborative Practice Competencies

The scoping review study by Poitras et al,^7^ points out that one of the benefits of telehealth regarding collaborative practice competencies is the improvement of interprofessional collaboration when well introduced to the team. What’s more, telehealth can facilitate information sharing between healthcare professionals and patients, which has a positive influence on interprofessional collaboration and patient engagement.^32^ According to Poitras et al,^7^ “*leveraging telehealth technologies not only bridges communication gaps among professionals but also fosters a cohesive approach to patient care, ultimately improving collaborative practice competencies across the team*.” Optimal use of technology improves communication between professionals and patients, which benefits continuity of care for patients with chronic diseases.^32^

## Theme 2: Challenges of Telehealth in Primary Care

### Variability in Patient Experience-Professional Relationships

The literature shows that people’s experience of telehealth varies greatly towards the challenges associated with its use in primary care. According to Cartwright et al,^33^ telehealth used without adequate infrastructure (eg, reliable digital platforms, dedicated telehealth equipment, technical support, standardized telehealth guidelines, etc.) increases the complexity for patients to develop a therapeutic relationship with certain professionals or to develop comfort with the use of technology. Indeed, patients refer to the diminished quality of the relationship with professionals in the telehealth context, giving the example of misinterpreting information received via telephone or video, or of disjointed communication during appointments.^4,33^ This can lead to inconsistency in the information received by the patient, as the non-verbal cues may be more difficult to assess during a telephone appointment or be perceived differently in a video consultation context.^31,34^ Furthermore, Brodar et al^35^ point out that telehealth work, if implemented without a clear structure, can lead to siloed work. This raises an important point because that goes against the principle of interprofessional collaboration. One of the causes of this siloed working, when using telehealth, is the fact that professionals work in locations geographically dislocated from other members of the interprofessional team with whom they would normally work face-to-face.^35,36^ Ultimately, this siloed practice can become detrimental to patients living with chronic diseases where the quality of care can be influenced by interprofessional collaboration.^35^

### Technological Barriers and Infrastructure Needs

In addition to the challenges outlined above, the lack of training in the use of telehealth is an issue frequently addressed in the scientific literature. Various studies highlight the need for support for professionals and patients to ensure collaborative practices in the telehealth context.^31,35^ For example, some studies mention the importance of training and support in the development of interprofessional collaboration skills in a telehealth context for professionals, patients, and families alike.^7,34,35^ Brodard et al^35^ point out that access to technological devices (computers, tablets, smartphones) is also an issue to be considered. Some studies mention that many patients and professionals are uncomfortable with the use of technology, which is detrimental to the quality of the consultation and the patient experience.^7^ This lack of technological infrastructure supporting patients and professionals is a condition that needs to be addressed in the healthcare system, especially from the perspective of interprofessional collaboration.^7^

## Theme 3: The Importance of Correctly Using Telehealth to Maintain Patient Engagement and Interprofessional Collaboration in Primary Care

### Patient Engagement and Satisfaction

Telehealth-related technology can support patients living with chronic diseases by promoting patient engagement and interprofessional working.^35,37,38^ Some authors support the fact that quality and satisfaction of care are neither affected nor different for patients who consult via telehealth or face-to-face.^3,5^ However, other research shows that the quality of care received during a telehealth consultation can be greatly affected if it is used inappropriately during a first appointment,^7^ or during a mental health appointment where the relationship between patient and professional is not yet well established.^33^ For example, it is mentioned by both patients and professionals that the therapeutic relationship is more difficult to establish during telephone appointments compared to video appointments.^31^ In addition, patients say they are more distracted because the time allotted for their consultation cannot be extended, since they are not in a conducive environment (eg, during a consultation during working hours or activities of daily living).^34,35,39^

### Advantages and Barriers of Telehealth in Primary Care

On the other hand, some authors agree that telehealth enables patients to receive care promptly.^35,37^ Some studies add that telehealth consultations reduce the costs associated with primary care^3,38^ and that access to healthcare professionals via telehealth is optimal in special situations (ie, pandemic, patients with travel difficulties, etc.).^31,35^ According to Hardcastle and Ogbogu,^36^ patients living with chronic diseases who have access to care feel a sense of security, which increases their satisfaction, maintains their commitment to their health and optimizes expected outcomes.^36^ Still, it is important to note that some authors mention the reduced interaction and different geographical locations of professionals, which makes interprofessional collaboration more complex in the telehealth context, due to inadequate use of telehealth.^3,35^ In addition, Breton et al^39^ highlight professionals’ concerns about the lack of structures needed to guide them in managing a patient’s care as a team (ie, how to enable interprofessional collaboration in a telehealth context). According to Poitras et al,^7^ developing telehealth skills remains as important as face-to-face consultations in establishing or optimizing interprofessional collaboration. That said, the same expertise needs to be developed at the level of professional teams, as well as for patients during telehealth consultations.^7^ One criterion for successful interprofessional collaboration in a telehealth context is the presence of a team leader who guides, trains, and facilitates the implementation for both professionals and patients.^7^ Finally, studies have shown that the technology used in telehealth consultations can increase stress and anxiety among healthcare personnel if they are not comfortable with its use,^4,7^ thus affecting interprofessional collaboration and patient engagement.

## Discussion

This paper aims to describe how patients living with chronic diseases describe their engagement in the interprofessional collaboration process in a telehealth context in primary care settings. While the search aimed to find evidence of collaborative processes involving both patients and professionals, most literature tends to document one perspective over the other, often missing the nuanced interaction between the 2. The rapid expansion of telehealth use in primary care and the presentation of the results enables us to make the following observation that it is crucial to prevent a regression to isolated practices. This could be achieved through the implementation of structured telehealth models supported by clear provincial guidelines and policies that detail the when, how, and why of telehealth use that could be enhanced by technologies that are used with no structure (no provincial guidelines, no policies detailing when, how, or why to use telehealth, etc.).^3,7,33-35,40^

While telehealth use began prior to the pandemic, it remains clear that significant gaps in understanding exist. The literature reflects diverse perspectives on patient satisfaction—some studies suggesting that telehealth is not inferior to face-to-face care,^3^ while others highlight difficulties in building trusting relationships due to disjointed communication and limited opportunities to express non-verbal communication.^33,38,39^ While telehealth offers numerous advantages, such as increased accessibility, time efficiency for patients, and cost-effectiveness for both patients and healthcare systems,^4,7,32,41^ it also presents challenges—such as siloed work, difficulty in maintaining patient connections, and a lack of tools to fully leverage its potential.^3,4,7,31,33,34,35^

These findings call attention to the pressing need for healthcare policies that guide the implementation of telehealth in primary care, ensuring that it fosters integrated care models rather than sustaining isolated practices. Policymakers should focus on developing frameworks that support interprofessional collaboration, and patient engagement providing necessary resources for technological infrastructure, standardized practices, and the training of both patients and healthcare professionals. In addition, policies should address barriers to telehealth access, such as geographic disparities, digital literacy, and the availability of technological devices^7,33,35^

## Next Steps

Based on these results, we are developing a comprehensive project aimed at addressing the literature gap on patient perspective regarding teleconsultation. This project will explore the nuances of interprofessional collaboration within telehealth setting in primary care, with a particular focus on recognizing and incorporating patient engagement in the collaborative process. This will provide several recommendations to the different establishments, decision-makers, and professionals to promote patient engagement, and interprofessional collaboration in primary care in a telehealth context.

## Limitations

Our scoping review has some limitations. Our choice of research strategy may have influenced the articles we captured because of the number of concepts chosen. On the other hand, having these specific concepts might have positively narrowed the research. In addition, the articles were limited from January 2002 until September 2023, also being a choice of the team to better structure the research subject. This timeline also prevented the research team from being captivated by the learning curve and the technology modifications. Some new articles might have been missed due to the critical increase in the use of technology in telehealth following the outbreak of COVID-19. The research team could have chosen different databases to search, according to the domain of research. The database chosen for this scoping review did reflect some multidisciplinary databases and not only specific domains. Finally, having a clearer definition of patient engagement at the beginning of the scoping might have captured different articles.

## Conclusion

This scoping review emphasized the challenges when using telehealth in primary care, the advantages of interprofessional collaboration and patient engagement when using telehealth, and the importance of a suitable use to maintain interprofessional collaboration and the patient engaged in telehealth. This scoping review underscores the critical role of patient engagement in interprofessional collaboration within a telehealth context, emphasizing the need to understand and integrate patients’ perspectives on their own involvement in collaboration. The review ultimately highlights how telehealth can either facilitate or hinder patient engagement, underscoring its relevance and originality in exploring these dynamics. Our findings empower researchers, clinicians, and patients to take tangible steps toward improving the use of telehealth by engaging the patient in collaboration among remotely working professionals. Moreover, researchers can leverage from our key message to further explore patient engagement in the process of interprofessional collaboration in primary care in a telehealth context. As telehealth continues to be widely used in healthcare, there is a need for reflection on how to optimize this working paradigm (virtual) for the benefit of both patients regarding their engagement and professionals regarding interprofessional collaboration. Telehealth has now become a permanent mode of patient care. Therefore, it is imperative to enhance support for professionals and patients as they incorporate this new practice by assisting them with tools and furnishing them with suitable infrastructure.

## Supplemental Material

sj-docx-1-jpc-10.1177_21501319251333858 – Supplemental material for Chronic Disease Patients’ Engagement in Interprofessional Telehealth Collaboration in Primary Care: A Scoping Review Supplemental material, sj-docx-1-jpc-10.1177_21501319251333858 for Chronic Disease Patients’ Engagement in Interprofessional Telehealth Collaboration in Primary Care: A Scoping Review by Monica McGraw, Anaelle Morin, Vanessa Tremblay Vaillancourt, Marie-Eve Poitras, Yves Couturier, Isabelle Gaboury and Marie-Dominique Poirier in Journal of Primary Care & Community Health

## Statements and Declarations

**Ethical Considerations:** This scoping review does not involve people, no ethical approval is required.
