# Supplementary material for: Chronic Disease Patients’ Engagement in Interprofessional Telehealth Collaboration in Primary Care: A Scoping Review
Source: J Prim Care Community Health. 2025 Jun 6;16:21501319251333858. doi: 10.1177/21501319251333858 (PMC12144354; doi:10.1177/21501319251333858)
Supplement: sj-docx-1-jpc-10.1177_21501319251333858 – Supplemental material for Chronic Disease Patients’ Engagement in Interprofessional Telehealth Collaboration in Primary Care: A Scoping Review [file sj-docx-1-jpc-10.1177_21501319251333858.docx]

**Supplement online material**

**Research strategy**

| **Telehealth/ Télésanté** | "Tele"OR "digital*" OR "remote*" OR "video" OR "Ehealth" OR "e-health" OR "electronic health" OR "virtuality" OR "virtuals"OR ("virtual"OR "virtuality"OR "virtualization"OR "virtualized"OR "virtualizing"OR "virtuals"[All Fields]) OR ("virtual"OR "virtuality"OR "virtualization"OR "virtualized"OR "virtualizing"OR "virtuals") OR ("virtual"OR "virtuality"OR "virtualization"OR "virtualized"OR "virtualizing"OR "virtuals") OR ("virtual"OR "virtuality"OR "virtualization"OR "virtualized"OR "virtualizing"OR "virtuals") OR ("virtual"OR "virtuality"OR "virtualization"OR "virtualized"OR "virtualizing"OR "virtuals")) |
| --- | --- |
| AND |  |
| **Primary Care/ Soins santé primaires** | ("primary care"OR "family practice"OR "general practice"OR "physicians’ family"OR "primary healthcare"OR "primary"OR "primary care"OR "family"OR "general"OR "family health team*") |
| AND |  |
| **Chronic Diseases/ Maladie chronique** | ("chronic disease*"OR "respiratory"OR "cardiac"OR "cardiology"OR "hearth failure"OR "COPD"OR "stroke"OR "hypertension"OR "diabetes"OR "orthopedic"OR "hiv"OR "high risk obstetrics"OR "sleep"OR "public health"OR "obesity"OR ("sickel"AND "ell") OR "anemia"OR "joint replacement"OR "dermatology"OR "autoimmune"OR "rheumatology"OR "rheumatoid arthritis"OR "lupus"OR "CKD"OR "cystic fibrosis"OR "asthma") |
| AND |  |
| **Interprofessionnal Collaboration/ Collaboration interprofessionnelle** | ((("interprofession*"OR "interdisciplin*"OR "inter occupation*"OR "interoccupation*"OR "inter institut*"OR "inter sector*"OR "intersector*"OR "multi profession*"OR "multiprofession*"OR "multi institut*"OR "multiinstitut*"OR "multi agenc*"OR "multiagenc*"OR "multi sector*"OR "multisector*"OR "multiorganisation*"OR "multi-organisation"OR "multiorganization"OR "multidisciplin*"OR "interorganisation*"OR "inter organisation*"OR "interdepartment*"OR "team*") AND "collab*") OR "liais*"OR "cooperat*"OR "shared"OR "joint"OR "integrat*") |
| AND |  |
| **Patient Engagement/ Engagement des patients** | (("patient*"OR "communit*"OR "partner*"OR "collaborat*"OR "commitment*"OR "particip*"OR "involv*") AND "patient*") OR "communit*"OR "partner*"OR "collaborat*"OR "commitment*"OR "particp*"OR "involv*") |
